# Omega-3 LCPUFAs (Long-Chain Polyunsaturated Fatty Acids) and Reading: The Mediating Role of Auditory Processing and the Interactions Among PUFAs

**DOI:** 10.3390/biomedicines13102517

**Published:** 2025-10-15

**Authors:** Maria Luisa Lorusso, Francesca Borasio, Carlo Agostoni, Eva Marie-Louise Syren, Stefano Turolo, Mariagrazia Benassi, Roberto Bolzani, Antonio Salandi, Francesca Nicoli, Marilena Vecchi, Malida Franzoi, Federica Martinez, Daniela Traficante, Massimo Molteni

**Affiliations:** 1 Unit of Child Psychopathology, Scientific Institute IRCSS E. Medea, 23842 Bosisio Parini, Italy; antonio.salandi@lanostrafamiglia.it (A.S.); francesca.nicoli@lanostrafamiglia.it (F.N.); daniela.traficante@unicatt.it (D.T.); massimo.molteni@lanostrafamiglia.it (M.M.); 2 SC Pediatria-Immunoreumatologia, Fondazione IRCCS Ca’ Granda Ospedale Maggiore Policlinico, 20122 Milan, Italy; carlo.agostoni@unimi.it (C.A.); eva.syren@unimi.it (E.M.-L.S.); 3 Department of Clinical and Community Sciences, University of Milan, 20122 Milan, Italy; 4 SC Nephrology Dialysis and Pediatric Transplantation, Fondazione IRCCS Ca’ Granda Ospedale Maggiore Policlinico, 20122 Milan, Italy; stefano.turolo@policlinico.mi.it; 5 Department of Psychology, University of Bologna, Campus of Cesena, 40126 Bologna, Italy; mariagrazia.benassi@unibo.it (M.B.); roberto.bolzani@unibo.it (R.B.); 6 Scientific Institute IRCSS E. Medea, 31015 Conegliano, Italy; marilena.vecchi@lanostrafamiglia.it (M.V.); malida.franzoi@lanostrafamiglia.it (M.F.); federica.martinez@lanostrafamiglia.it (F.M.); 7 Department of Psychology, Catholic University of the Sacred Heart, 20122 Milan, Italy

**Keywords:** omega-3 long-chain poly-unsaturated fatty acids, EPA, DHA, ALA, reading, writing, mediation, moderation, visual processing, auditory processing

## Abstract

**Background**: The present study aimed to clarify the neurocognitive processes through which blood levels of omega-3 LCPUFAs affect reading and writing abilities. **Methods:** A total of 74 school-age children whose reading and writing skills varied from normal to largely below normal underwent an assessment of reading and writing abilities, auditory and visual processing, phonological awareness, attention, and executive functions. Exploratory factor analysis extracted three neuropsychological factors whose roles as mediators between omega-3 LCPUFAs and reading/writing abilities were tested in GLM mediation models. The possible interactions with other PUFAs were further investigated. **Results** (on 73 participants): Omega-3 LCPUFA levels (EPA and DHA) correlated with reading and writing abilities and with the three extracted factors. Auditory–phonological processing skills were found to be significant mediators of the effect of PUFAs (especially EPA) on reading and writing abilities, whereas DHA and AA/ALA significantly moderated some of these effects. **Conclusions:** The link between omega-3 LCPUFAs and reading and writing abilities seems to be mediated mainly by the effects of LCPUFAs on auditory–phonological processing skills. These effects are especially linked to EPA, but they are modulated by DHA and AA/ALA levels. Hypotheses about possible molecular mechanisms at the basis of these effects are discussed.

## 1. Introduction

Reading and writing are complex skills that involve many different neuropsychological functions. In addition to phonological processing, low-level visual and auditory perception and attention mechanisms as well as memory and executive functions have been called into play in explaining reading and spelling disorders [1,2,3], and fatty acid (FA) metabolism seems to be one of the possible underlying factors [4,5,6].

Omega-3 long-chain polyunsaturated fatty acids (LCPUFAs), such as DHA (docosahexaenoic acid, 22:6 *n*-3) and EPA (eicosapentaenoic acid, 20:5 *n*-3), play a crucial role in brain health. These nutrients, found mainly in marine sources and plant oils, have been shown to influence fetal brain development, exerting their effects on microglial activity, neuroinflammatory regulation, and synaptic plasticity [7]. DHA is present in highest concentration in brain cells, where it is located across the inner and outer leaflets of the cell membrane, improving neuronal electrical activity and synaptic development [8,9]. DHA is essential for prenatal and early postnatal brain and visual development and for the functioning and maintenance of the visual and nervous systems [10,11]. EPA, moreover, plays a key role in the production of a wide range of essential cytokines [8,9]. Some studies have suggested that abnormalities in fatty acid metabolism may contribute to different neurodevelopmental disorders, including ADHD (attention-deficit hyperactivity disorder) and developmental dyslexia (DD) [12,13]. Indeed, children with neurodevelopmental disorders often exhibit reduced levels of omega-3 LCPUFAs, particularly DHA and EPA, corresponding to increased production of proinflammatory cytokines linked to omega-6 PUFAs metabolism [6,14]. DHA and EPA modulate oxidative stress and free radical generation, thus contributing to the reduction of inflammatory processes. Inflammatory cytokines, in turn, have a negative impact on the prefrontal cortex, controlling executive functions [7,8,9]. Each of these omega-3 LCPUFAs has specific effects. DHA, the most abundant omega-3 fatty acid in the brain, supports cognitive processing, neurite outgrowth, synaptic communication, and membrane fluidity while fostering neuronal survival. Deficiencies in alpha linolenic acid (ALA) may result in reduced concentrations of DHA in the retina and in the occipital cortex, affecting visual processing [15]. Several studies have shown a positive effect of DHA in favoring morphological development of neural cells. Studies on cultured rat and mouse hippocampal neurons have shown that culture medium supplemented with DHA enhances neurite length per neuron, the number of branches per neuron [16], and dendritic arborization complexity [17]. More specifically, the mechanism that appeared to be impaired in DHA-depleted hippocampi was long-term potentiation (LTP), a special form of synaptic plasticity involved in learning and memory [16], along with reduced expression of glutamate receptors that may cause inadequate glutamatergic synaptic transmission. A meta-analysis and a clinical trial [18] suggested that EPA is more effective than DHA in reducing symptoms and improving cognition in children with ADHD, possibly linked to the anti-inflammatory properties of EPA and its derivatives (different from DHA), and more evidently in children who start from low baseline levels. However, other authors [19] have argued that DHA may play an even more important role than EPA in supporting the development of reading and spelling abilities. The link between DHA and reading would especially have to be found in magnocellular functions. Indeed, lack of omega-3 LCPUFAs—especially DHA—has been called into play in the hypothesis that DD is caused by a dysfunctional magnocellular system related to the visual [20,21] and auditory modalities, which can be related to a variable degree [22]. Magnocellular neurons belonging to the “dorsal” visual system are very sensitive to visual motion and to the timing of visual events, but they do not really contribute to the identification of form and details [20,21,23]. In addition, the auditory system contains magnocellular neurons analogous to visual neurons [21,22]. This system is organized in a manner similar to the visual magnocellular system and participates in accurate auditory sequencing and timing. The left superior temporal cortex also receives auditory information about the sequence of phonemes in spoken language and probably integrates the various sources of information, providing the basis of grapheme/phoneme associations necessary to consolidate the phonological and metaphonological skills involved in reading and spelling or writing to dictation. In both cases, the role of the magnocellular system would be more evident for reading and writing through the sublexical (especially necessary for novel word or infrequent word (de)coding) route than through the lexical route (especially involved in real word (de)coding) [24].

Although it is generally acknowledged that marine *n*-3 fatty acids exhibit greater biological activity than their plant-derived counterparts [25], emerging evidence highlights the potential of ALA in improving cognitive function and supporting brain health [26]. A 16-weeks supplementation of omega-3 PUFAs from linseed oil was shown to improve spatial memory, reduce inflammatory markers (TNF-a), and decrease toxic metabolite levels in the central nervous system in mice [27], but ALA also improved lexical fluency in adult humans [28] after 12 weeks, possibly as a consequence of enhanced neuron cell membrane fluidity and improved intercellular connectivity. A crucial and exclusive role of ALA in reducing the decline in global cognitive function and memory has been highlighted by a recent Dutch study in a large sample of older adults [29]. It has been suggested that ALA may have a role in actively reducing inflammation through the effects of resolvins and protectins, molecules that are generated from omega-3 PUFA precursors [30].

Altogether, then, it appears that omega-3 LCPUFAs participate in general neuronal health and growth and, more specifically, affect visual and auditory processing and possibly executive functions through their effects on the magnocellular systems for the visual and auditory modalities, occipital areas, the hippocampus, and frontal/prefrontal regions.

The differential effects and potential interactions of different LCPUFAs and, possibly, of other omega-3 PUFAs are still in the process of being clarified and are very difficult to anticipate in a relatively less studied domain such as the domain of reading and spelling, which, in turn, reflects the contribution of several different processes encompassing visual, auditory, and executive functions. It should be remembered that omega-3 PUFAs, beyond their specific effects (as highlighted above) on neural structures and systems, exert a general anti-inflammatory effect both through eicosanoid production and though a reduction in the release of proinflammatory cytokines, such as interleukin-1ß (IL-1ß), -2 (IL-2), and -6 (IL-6); IFN-; and TNF- [25]. These cytokines, in turn, can affect the brain systems through different mechanisms: on the one hand, reducing the availability of neurotransmitter precursors, influencing their metabolism, transport, and regulation, and, on the other hand, impacting the HPA (hypothalamic–pituitary–adrenal) axis (the neuroendocrine system that regulates the body’s response to stress by controlling the release of hormones) as well as mRNA-encoding proteins involved in neurotransmitter metabolism [25].

**Figure 1:**
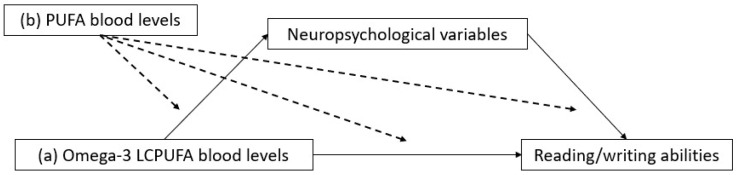
General mediation/moderation model under investigation.

The working hypotheses for the present study consequently were (i) that the effect of omega-3 LCPUFAs on reading ability would be mediated by neuropsychological variables related to auditory–phonological, visual–perceptual, or visual–attentional skills; (ii) that different omega-3 LCPUFAs could be selectively influencing reading speed, reading accuracy, and writing accuracy due to their different types of actions on brain cells and neural systems; and (iii) that their action could be exerted as either (a) independent predictors of reading ability through the mediation of neuropsychological variables or (b) moderators of the path linking other LCPUFAs to reading ability. These three possibilities are illustrated in Figure 1. Under hypothesis (iii-b), different omega-3 LCPUFAs could act synergistically or inhibit each other’s effect, and the possibility is also investigated that the AA/ALA ratio (arachidonic acid, 20:4 ω-6/α-linolenic acid, 18:3 ω-3), which was found to be especially crucial in previous analyses related to the same study [4,5], modulates the effect of omega-3 LCPUFAs.

## 2. Materials and Methods

### 2.1. Participants

A total of 74 children, aged between 8.17 and 14.17 years (M = 10.87; SD = 1.47; female = 42, male = 32), participated in this study. In the whole group of children, 57 were diagnosed with developmental dyslexia (DD) according to ICD-10 diagnostic criteria, while 17 were normally reading children. Participants with reading disorders were recruited at the child neuropsychiatry units of the recruiting centers in northern Italy, while TD children were recruited from the same geographic area among the schoolmates and friends of the children with DD. All participants had to fulfill the following inclusion criteria: (a) age between 7 and 15 years and attending at least the third grade of primary school; (b) IQ ≥ 80; (c) monolingual speakers or bilingual speakers with perfect (native-like) mastery of the Italian language. Additional inclusion/exclusion criteria for children with DD were (a) having been previously diagnosed with DD based on standard inclusion/exclusion criteria [31]; (b) absence of comorbidity with ADHD and other neuropsychiatric or psychopathological conditions (comorbidity with other learning disorders was allowed); and (c) not having received neuropsychological treatment for DD before. Inclusion/exclusion criteria for TD children were (a) normal school achievement as reported by teachers and parents and (b) no z-scores below −1.5 with respect to age mean in word and nonword reading tests and in tests of writing to dictation (DDE-2 battery) [32]. Overall, the group of children included in this study represent a continuous distribution of reading and writing abilities as well as neuropsychological functions related to reading and spelling. Therefore, the children were handled as a single group for the purposes of this study. Written informed parental consent was obtained before the start of this study. This study was approved by the Ethical Committee of Scientific Institute IRCCS E. Medea in accordance with the European Union’s Standards of Good Clinical Practice and the Declaration of Helsinki on 12 December 2019 (protocol number 76/19-CE).

### 2.2. Measures and Procedure

The present study is part of an ongoing project on the efficacy of omega-3 and omega-6 LCPUFA supplementation to enhance the effects of neuropsychological treatment in children diagnosed with DD (registered in ClinicalTrials.gov, Code NCT04287530). Only a subset of measures was taken into account for this study, selected on the a priori hypotheses linking omega-3 LCPUFAs to auditory–phonological, visual, and executive neurocognitive processes involved in academic skills.

All measures were administered individually by trained psychologists in one session of 90 min during the pre-test assessment of the main project. Blood samples were obtained by collecting a drop of blood from a fingertip, following the methodology described by Marangoni et al. [33] and also Borasio et al. [4,5]. The dried blood spot was methylated with HCl/MeOH (Supelco, MERCK), and fatty acid methyl esters (FAME) were extracted with hexane and injected into a Shimadzu Nexis GC-2030 gas chromatograph. A 30 m capillary column (FAMEWAX, RESTEK) was used to separate the FAME. The Labsolution software v.5.97 SP1 (Shimadzu) was used to identify FA species using the retention time of standards (PUFA1, PUFA2, PUFA3 (Supelco, MERCK); and NHI-F (AccuStandard, RESTEK)). Single fatty acids were identified based on predetermined standards and on the time needed by each molecule to reach the detector.

In the present study, EPA and DHA were considered as measures of interest based on previous literature, while the AA/ALA ratio was added as a potential moderator based on previous results [4,5].

Neuropsychological tests commonly employed in the assessment of reading disorders in Italy were used. Specifically, the following tests were conducted.

- Cognitive measures. The Wechsler Intelligence Scale for Children, Fourth Edition (WISC-IV) [34] and the Raven’s Colored Progressive Matrices Test (CPM) [35,36] were adopted.
- Single word/nonword reading. “DDE-2: Batteria per la Valutazione della Dislessia e Disortografia Evolutiva-2” (Assessment battery for Developmental Reading and Spelling Disorders-2) [32] was used to assess speed and accuracy (expressed in the number of errors) in reading word and nonword lists (112 words and 48 nonwords in total).
- Word and sentence writing to dictation. Two dictation tasks were taken from the DDE-2 battery [32], giving accuracy scores (number of errors) in writing (48) words and (12) sentences.
- Phonological awareness. The NEPSY-II test [37] was subdivided into two parts. In the first part, the child is required to establish a correspondence between sounds and images and to select the corresponding image based on a partial sound. The second part of the test requires the child to encode a verbal stimulus and manipulate its phonetic structure by either removing a sound or replacing one sound with another. Raw scores were calculated as the number of correct answers (range, 0–53). Cossu’s test [38] consists of two subtests. In the (b) Phonemic Elision task, the child is asked to recognize and isolate the phonemic constituents of 20 words and report the nonword resulting from deleting the first two phonemes in the word read by the examiner. (c) The Phoneme Blending task assesses the capacity to derive a phonemic pattern from distinct phonemic units. The examiner presents 20 words letter by letter, and the child is required to identify and report the resulting word. For Cossu’s tasks, the scores refer to the total number of errors.
- Rapid automatized naming. “Denominazione Rapida” (rapid automatized naming test—RAN) [39] was used to assess the naming speed for familiar stimuli (colors). Two matrices (10 rows of five stimuli each) were presented, and the child was asked to sequentially name each visual stimulus of the matrix as quickly and accurately as possible. Two raw scores were recorded: speed (expressed in seconds) and accuracy (expressed as the number of naming errors).
- Visual search. “Ricerca visiva” (visual search—VS) [39] was used to assess speed and accuracy in visual search for familiar stimuli (digits). Two matrices (10 rows of five stimuli each) were presented, and the child was requested to cancel one of the stimuli (the number “7”) presented in the matrix as quickly and as accurately as possible. Two raw scores were recorded: speed (expressed in seconds) and accuracy (expressed as the number of cancellation errors).
- Rhythmic pattern discrimination. This procedure was proposed by Cantiani et al. [40]. The children were required to discriminate and recognize four-tone rhythmic patterns. The children listened to the pairs of rhythms and were instructed to indicate, by an oral answer, whether the two rhythms were equal or different. The total number of correct answers (pattern total) was also analyzed.
- Inhibition in visual–spatial attention (VSA). The flanker (FL) test was taken from the Amsterdam Neuropsychological Tasks (ANT) [41]. In this task, a central target stimulus surrounded by eight distractors (flankers) is presented in the middle of the screen. The color of the target stimulus is linked to the answer button: if it is blue, the left button must be pressed; if it is yellow, the right button should be pressed. The flanker task consists of two parts. Part 1 consists of 40 trials with compatible flankers (the same color as the central stimulus) and neutral flankers (the color is different from the color of the central stimulus). Part 2 consists of 80 trials with the target stimulus surrounded by compatible or incompatible flankers (color associated with the opposite response). In Part 2, the children are required to answer exactly as required in Part 1, pressing the left button if the target stimulus is blue or pressing the right button if the target stimulus is yellow. The flanker effect (indicating the ability to suppress irrelevant information and focus attention) was calculated by subtracting the mean reaction time in compatible trials from the mean reaction time in incompatible trials. Moreover, flanker facilitation was calculated by subtracting the mean reaction time on compatible trials of part 2 from the mean reaction time on neutral tasks in part 1.
- Shifting attentional visual task (SAV). This task from the ANT battery [41] was used to assess attentional flexibility. A colored square jumps randomly on a horizontal bar to the right or left, and depending on the color of the square immediately after the jump, the subject has to execute a compatible response (press the key toward where the jump was directed) or an incompatible response (‘mirror’ movement; press the right key when the jump was to the left and vice versa). The task consists of three parts. In the first part, the color of the square is constant, and the child is asked to execute a compatible response after the square jump. In the second part, the color of the square is constant, and the child is asked to execute an incompatible response. In the third part, the color varies, requiring attentional flexibility by continuously adjusting the response type. The post-response interval (PRI, i.e., the time between response execution and next stimulus onset) is 250 ms in all parts. The first two parts consisted of 40 trials, whereas the third part consisted of 80 trials. The SAV congruency effect was calculated by subtracting the mean RT (in the third part of the test) of compatible responses from the mean RT of incompatible responses. Instead, SAV rule complexity was calculated by subtracting the mean reaction time of incompatible responses in the second part of the test from the mean reaction time of incompatible responses in the third part.
- Cue effects in VSA. A classical Posner’s cueing paradigm for measuring covert visual–spatial attention (VSA) was constructed following the same procedure described by Facoetti et al. [42]. The fixation point consisted of a cross appearing at the center of the computer screen, and two circles were presented peripherally: one to the left and one to the right of the fixation point. The target dot was preceded by a spatial cue (1.58° arrow appearing in the center or periphery), which could be valid (80% of the trials) or invalid (20% of the trials). Children were instructed to keep their eyes at the fixation point throughout the duration of the experimental session. Each trial started with the onset of the fixation point, accompanied by a 1000 Hz warning signal tone. After 500 ms, the two circles were displayed peripherally, and 500 ms later, the cue was shown for 50 ms. After 300 ms, the target appeared for 50 ms inside one of the two circles, while in valid trials, the target was presented inside the circle pointed by the arrow cue, whereas in invalid trials, the target appeared in the circle on the opposite side. The children were instructed to react as quickly as possible by pressing the spacebar on the computer keyboard if the target dot appeared inside the circle. The cue effect was calculated by subtracting the mean reaction time in valid condition trials from the mean reaction time in invalid condition trials. The left–right difference in the VSA cue effect was computed by subtracting the cue effect in the right visual field from that in the left visual field. Left–right differences in speed for the VSA cue effect were calculated (VSA left−right cue effect).
- Motion coherence. Motion perception was evaluated using the Motion Coherence test described by Benassi et al. [43,44]. The stimulus consisted of 150 high-luminance dots (luminance = 51.0 cd/m^2^, dot diameter = 3 arcmin, dot density = 1.25 dots/deg^2^) that could either move coherently at a constant speed (1.5 deg/s) in one of eight directions in space (four cardinal and four oblique) or in a Brownian manner (noise dots) within a circular frame of 6.2 deg on a black background (0.2 cd/m^2^). The present test consisted of high-temporal-frequency stimuli that moved across the screen and required the children to identify the direction in which the coherent dots were moving. The stimulus was presented on the screen for 1000 ms; then, it disappeared, and all eight possible directions appeared on the screen, as indicated by eight grey arrows. The children were required to indicate the direction of the coherent moving dots by clicking the mouse on the indicated arrow. The test consisted of five levels corresponding to five levels of coherence, and each level was composed of eight trials. The first level started from the condition of 100% coherence (all dots moved coherently in one specific direction; no noise), and at each level, the coherence rate decreased by 2 dB (100%, 63.10%, 39.81%, 25.12%, and 15.85%). The global score was calculated as the percentage of correct responses.
- Gratings. This task has been described by Wilmer et al. [45]. The targets were vertical (90°) and sinusoidal gratings with a spatial frequency of 0.5 cycles per degree. For tasks, temporal frequency ranged from 2.5 to 7.5 Hz (temporal frequency = spatial frequency × velocity). The gratings were shown on a computer monitor through a circular window subtending 19° of visual angle with a space average luminance of 45 cd/m2. The duration of each stimulus was 300 ms, and the interval between the two comparison stimuli was 500 ms. The children were asked to indicate which of the two gratings was moving faster. Two comparisons were used: the easy comparison (15 vs. 5 °/s) involved a difference of 100%, and the more difficult comparison involved a difference of 20%. The percentage of correct responses for each of the 24 items was recorded.

In addition to these measures, the larger study protocol included a text reading test, an inhibitory ability test, and a tactile finger localization test, which were not considered in the present study.

### 2.3. Data Analysis

Data of one child with DD in the SAV task were missing due to technical problems during data collection and recording. Consequently, all subsequent analyses were performed on a group of 73 children.

Power/sensitivity analysis was conducted using the G*Power software (v. 3.1.9.2) [46]. First, the power analysis conducted for correlations (exact tests, bivariate normal model) showed that 42–43 participants are required to reach a power of 0.80 with an effect size of approximately 0.40 (two-tailed), corresponding to the average effect size found in previous studies. As to mediation, in the absence of previous reference data, a sensitivity analysis (conducted with linear multiple regression, fixed model, single regression coefficient with four predictors, and two-tailed *t*-test) [47] showed that 73 participants would allow the detection of an effect size of f2 = 0.11 (corresponding to a low to medium effect size) with a power of 0.80.

Regarding moderation, sensitivity analysis showed that 73 participants would allow for the detection of a moderation effect (linear multiple regression, fixed model, F-test for R2 increase with two tested predictors and three to six total predictors) of f2 = 0.138 (in the low range of the average effect size). On these grounds, a sample size of 73 participants was deemed sufficient to detect low-to-medium effect sizes in the mediation and moderation models.

First of all, an exploratory factor analysis was applied to reduce the number of neuropsychological variables. A 3-factor solution emerged from “minimal residual” extraction with Oblimin rotation (allowing for non-orthogonal factors to be extracted). The results of this factor analysis are reported in Table 1 (only saturations ≥ 0.25 are reported).

The three factors were identified with the following constructs: Factor 1 as “Auditory Processing” (AP), where the visual search accuracy component may be thought to represent a perceptual precision aspect, and the Pattern accuracy component to represent the non-phonological, auditory processing contribution; Factor 2 as “Visual–Perceptual” (VP) (including the tests related to the M-visual system and with the Visual search speed component likely to support the link with the RAN color test speed); and Factor 3 as “Visual–Attentional” (VA). AP turned out to be correlated with both VP (rho = 0.417, *p* < 0.001) and VA (rho = −0.269, *p* = 0.022), whereas VP and VA were not mutually correlated.

For all the analyses, three general scores were computed for reading and writing measures based on raw scores: (1) DDE general reading speed score, i.e., the average of speed scores (secs) for word and nonword reading; (2) DDE general reading accuracy score, i.e., the average of accuracy scores (errors) in word and nonword reading; and (3) DDE general writing accuracy scores, i.e., the average of accuracy scores (errors) in word and sentence dictation. The three scores were all highly intercorrelated (as shown in Table A1).

Next, correlational analyses were performed considering the entire sample of children (n = 73). Due to the presence of outliers in the reading, writing, and neuropsychological tasks and in the resulting factors as well as in the LCPUFA levels, nonparametric correlations were used.

Standard GLM Mediation models [48,49] were adopted to test the possible mediating role of neuropsychological functions (those significantly associated with reading/writing performance) in the relationship between PUFAs and reading/writing skills. Raw scores were used for all measures in the mediation models, and age was entered into the models as a covariate. No Bonferroni correction was applied considering the mutual correlations among all variables used for the analyses and the a priori structure of the study design.

The GLM models were constructed based on the patterns of previously observed correlations. Specifically, triplets of intercorrelated variables, including one PUFA variable, one neuropsychological factor, and one reading/writing variable, were identified in order to construct and test plausible and meaningful mediation and moderation models. Two different PUFAs (EPA and DHA), three neuropsychological variables (the three factors identified through factor analysis), and three reading/writing indicators (writing accuracy, reading accuracy, and reading speed) were considered. Additional PUFA variables, namely, the remaining omega-3 LCPUFA (either DHA or EPA) that did not prove to be a predictor in the model or the AA/ALA ratio (independent of both EPA and DHA and not correlated with any of the emerging factors but significantly related to reading speed measures) that was shown to be a sensitive indicator in previous phases of the study [4,5], were tested as potential moderators in the models where EPA or DHA were found to have significant (direct or mediated) effects on reading and/or writing.

## 3. Results

### 3.1. Descriptive Statistics

The characteristics of the entire sample of included children (N = 73) are presented in Table 2.

### 3.2. Results of Correlations

As shown in the correlation tables reported in Appendix A, there are correlations linking EPA and DHA with all reading and writing scores. Similarly, there were significant correlations between the two omega-3 LCPUFAs and the three neuropsychological factors; more precisely, EPA correlated with AP and VP (and almost significantly with VA), while DHA significantly correlated with VA (see Table A3). Finally, the correlations between the three reading and writing variables and the three factors were all significant (see Table A1).

### 3.3. Results of Mediation and Moderation Analyses

Based on the results of the correlational analyses, triplets of variables that were found to be mutually linked were used in mediation and moderation models.

**Figure 2:**
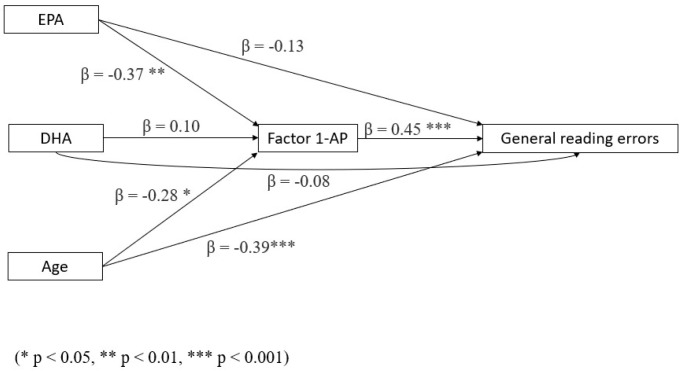
Mediation model with general reading errors as a dependent variable, AP as a mediator, and age and omega-3 LCPUFA levels as covariates (N = 73).

**Figure 3:**
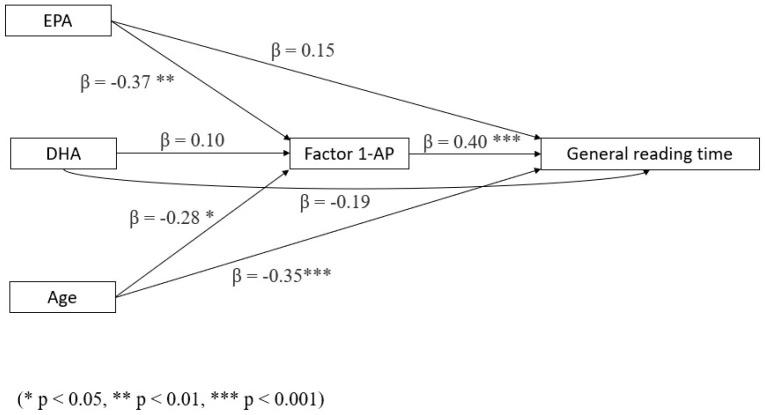
Mediation model with general reading time as a dependent variable, AP as a mediator, and age and omega-3 LCPUFA levels as covariates (N = 73).

**Figure 4:**
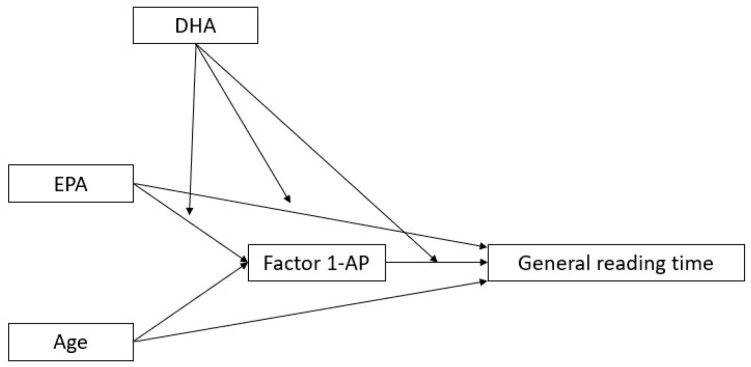
Moderated mediation model with general reading time as a dependent variable, AP as a mediator, DHA as a moderator, and age and EPA as covariates (N = 73).

Mediation analyses gave the following results:

1. EPA, but not DHA, has a negative indirect effect on general reading errors (i.e., errors decrease with increasing EPA) mediated by AP (see Figure 2 and Table 3).

2. EPA has a significant indirect negative effect (i.e., a reduction in reading time) mediated by AP and a direct (non-significant but with an equal, opposite beta coefficient), positive (detrimental) effect on general reading time; for this reason, the total model is not significant for EPA (see Figure 3 and Table 4 for full details).

3. Moreover, a moderation effect for DHA on the direct link between EPA and reading time emerged, showing that the direct (positive, hence detrimental) effect of EPA on reading time is “neutralized” at high levels of DHA (see Figure 4 and Table 5 for full details).

**Figure 5:**
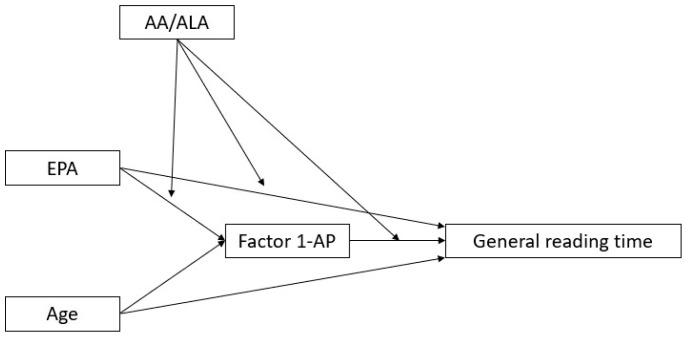
Mediation model with general reading time as a dependent variable, AP as a mediator, AA/ALA as a moderator, and age and EPA as covariates (N = 73).

Additionally, AA/ALA moderated the direct effect of EPA on reading time, strongly decreasing it. In fact, the direct effect of EPA was large and significant (negative) at low AA/ALA levels, non-significant at average AA/ALA levels, and non-significant but reversed (positive) at high AA/ALA levels. In contrast, the indirect effect of EPA through AP was always negative (favorable) and significant, regardless of AA/ALA levels. As a result, the total (and advantageous) effect of EPA on reading time originating from the sum of the two effects, direct and indirect, was only significant at low and average levels of AA/ALA (see Figure 5 and Table 6 for full details).

**Figure 6:**
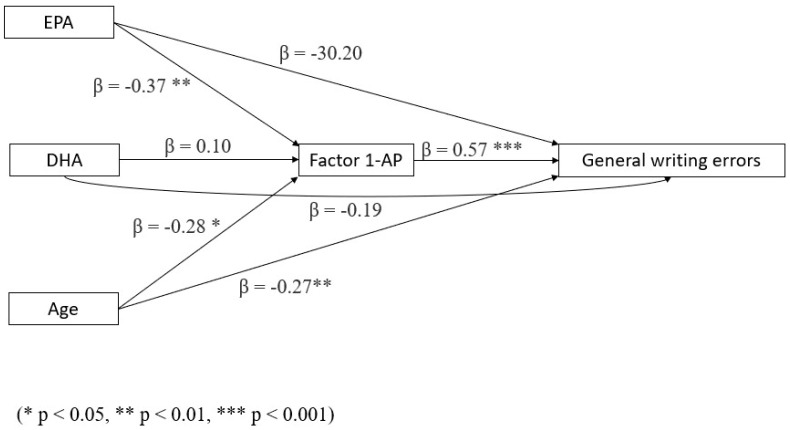
Mediation model with general writing errors as a dependent variable, AP as a mediator, and age and omega-3 LCPUFA levels as covariates (N = 73).

4. Regarding writing accuracy (general writing errors), AP was found to significantly mediate the indirect effect of EPA (but not DHA) on writing errors, whereas DHA (but not EPA) showed a (close to) significant negative direct effect. The total effect of both omega-3 LCPUFAs failed to reach significance. In the case of DHA, this happened because the negative (advantageous) direct effect was counterbalanced by a positive (disadvantageous but non-significant) indirect effect; in the case of EPA, the indirect effect was too small, and no direct effect emerged at all (although the total effect for EPA was close to significance, *p* = 0.062) (see Figure 6 and Table 7). No significant moderation effect of DHA emerged for the indirect effect of EPA on writing errors through AP (while AA/ALA, as shown for reading time, moderates the indirect effect of EPA on AP). No other significant mediation or moderation effects were observed.

## 4. Discussion

The results of the present study allow for the formulation of some preliminary hypotheses about the specific role of different omega-3 LCPUFAs in reading and writing abilities in children. Factor analysis applied to the numerous neuropsychological variables highlighted the existence of three factors that were identified as auditory processing (AP), visual–perceptual processing (VP), and visual–attentional processes (VA). EPA was found to correlate with AP and VP (and almost significantly with VA), while DHA was significantly correlated with VA (see Table A1 in the Appendix A). The three factors all correlated with all of the learning variables: reading accuracy, reading speed, and writing accuracy (see Table A2 in the Appendix A).

These correlations confirm the overall positive influence of DHA and EPA on learning processes [6,12,13,14,18,19]. Even if both are involved in accuracy mechanisms (i.e., in reducing reading and writing errors), EPA (but not DHA) is also involved in reading speed. The differential effects of DHA and EPA on reading have rarely been addressed, and they are usually considered together. However, some studies have highlighted significant differences in their neurobiological effects [50,51], possibly also mediated by their bioactive derivatives [9].

Considering the working hypotheses of this study, hypothesis (i), that the effect of omega-3 LCPUFAs on reading ability would be mediated by phonological, visual–perceptual, or visual–attentional skills, is only partially confirmed. Indeed, despite the many correlations linking the different neuropsychological factors with omega-3 LCPUFAs on the one hand and with learning abilities on the other hand, only phonological and auditory processing skills emerged as mediating mechanisms between PUFA levels and reading and writing abilities. Hypothesis (ii), that different omega-3 LCPUFAs could be influencing reading speed, reading accuracy, and writing accuracy, is also confirmed. Among omega-3 LCPUFAs, EPA seems to have a prominent role, while DHA and AA/ALA appear to exert a moderating effect on its action. This centrality of EPA and AA/ALA in affecting reading abilities had already been pointed out by Cyhlarova and colleagues [52].

Hypothesis (iii), that the action of LCPUFAs could be exerted as either (a) independent predictors of reading ability through the mediation of neuropsychological variables or (b) moderators of the path linking other LCPUFAs or neuropsychological variables to reading ability, has led to a complex pattern of mutual relationships. Mediation models clearly show that EPA, but not DHA, contributes to decreasing general reading errors through the mediation of AP, whereas there is no significant direct effect of EPA on reading accuracy. EPA also had an indirect, negative (enhancing speed) effect mediated by AP (along with an additional direct, positive, and hence detrimental effect) on general reading time. Regarding writing accuracy, there was an indirect effect of EPA, which was again mediated by AP, and a direct (but not fully significant) effect of DHA. Both effects are advantageous in reducing writing errors. Indeed, some of the well-studied effects of DHA on visual functions (especially those on the retina but also those on synaptic vesicles reaction to action potentials, enhancing the speed and efficiency of neuronal signaling [53]) could be expected to be direct rather than mediated by higher-order neuropsychological functions. On the other hand, the simultaneous presence of detrimental and favorable effects for the same fatty acid is not surprising and has been previously described, e.g., [54]. For instance, a recent comprehensive review [55] on the effects of omega-3 PUFAs on the auditory system highlights that both deficiency and excessive intake of omega-3 LCPUFAs, particularly DHA, can lead to auditory neural conduction impairment and reduced hearing acuity. It has been shown in studies on rats that inadequate dietary omega-3 FAs decrease dopamine levels and 5-hydroxyindolacetic acid (5-HIAA) in the lateral lemniscus and inferior colliculus, slowing nerve conduction in the auditory pathway, which is reflected in the ABR wave [56]. These findings could be due to difficulties in myelinization (e.g., delayed myelin deposition or instability in myelin composition) and synaptic in the auditory pathway. Hearing thresholds can also be affected: indeed, both deficient and excess intake of DHA, LA, and ALA during pregnancy and lactation can worsen hearing thresholds, especially at high frequencies. Particularly, DHA is described as having a relevant role in the neurodevelopment of auditory structures within the brainstem, possibly related to nerve repair or to antioxidant properties [55]. While what emerges from animal studies suggests that a deficiency in omega-3 PUFAs can lead to auditory impairments, it is difficult to determine the correct dose for optimal neurodevelopment of the auditory system, as high levels of omega-3 intake can also harm it, possibly through a reduction in AA levels, increased oxidative stress and cell apoptosis, and/or hormonal changes. It has further been suggested that the benefits of DHA supplementation on cognitive development are specific to cases of DHA deficiency [57].

Within a more neuropsychology-oriented framework, Laasonen and colleagues [58] had found a positive association of EPA and DHA with temporal processing acuity (TPA) in children with oral cleft; however, other studies, such as, e.g., Hurtado et al. [59], found that supplementation of omega-3 LCPUFAs during pregnancy and lactation does influence both mother’s and newborn’s FA profile but does not affect visual and cognitive/psychomotor development up to 12 months. Altogether, it seems that the effects of LCPUFA supplementation on auditory functions are dose-dependent, age-dependent, as well as population-dependent.

The finding of contrasting effects, in the positive and in the negative direction, for EPA and, in general, for omega-3 PUFAs is not surprising considering mixed results reported in the literature. However, the picture emerging from the present results also suggests that different neurobiological mechanisms are involved in the direct effect of PUFAs on learning abilities and the indirect effects exerted through the mediation of neuropsychological factors and systems, sometimes acting in the opposite direction. This implies that any hypothesis about the exact mechanisms involved is at a still very speculative level and that further systematic research on typically and atypically developing populations is badly needed.

Interesting results emerged from the moderation analysis that tested the existence of possible interactions among PUFAs related to reading ability. More specifically, it was found that DHA moderates the direct effect between EPA and reading time. At high levels of DHA, the detrimental direct effect of EPA on reading time disappears, allowing the indirect effect (although also slightly reduced by DHA increase) to express its benefits on reading speed. EPA and DHA thus interact in a positive way on reading speed, although they show some antagonistic effects. Research on interactions between DHA and EPA is scarce, but a recent study [60] showed that adding EPA to DHA in a supplementation program with rats did not lead to lower DHA levels as expected but rather to similar DHA levels. On the other hand, a DHA-rich diet also increased EPA levels. The authors hypothesized two possible mechanisms: one is the retroconversion from DHA to EPA, and the other one is the slowing of EPA metabolism as a consequence of high DHA levels, leading to the accumulation of EPA in plasma and organs, in agreement with previous reports indicating that dietary intake of a DHA supplement increases brain DHA but also leads to higher cerebral levels of other omega-3 PUFAs. This suggests that the presence of DHA could have potentiated the effects of EPA by increasing its presence in the brain.

The effect of the AA/ALA ratio (whose components do not belong to the omega-3 LCPUFAs group) as a moderator of the effects of EPA on reading speed in the present study is multifaceted. Indeed, with increasing AA/ALA, the direct effect of EPA increases and shifts from favorable to unfavorable, while the favorable indirect effect through AP does not change. Thus, a higher proportion of ALA with respect to AA increases EPA’s benefits on reading speed. These results partially confirm the data on the positive role of omega-3 FAs (including ALA) in cognitive efficiency [52,61]. Indeed, the effects of ALA on cognition are scarcely studied but seem to be multifaceted [61], from support to neurogenesis and neuronal preservation through the inhibition of glutamatergic transmission and activation of potassium channels (enhancing synaptic functions) and to immune as well as epigenetic processes [62]. ALA was also found to have anti-inflammatory, neuroprotective, and antidepressant effects [10,11], and it was further shown to reduce neuronal apoptosis and the toxic effects of heavy metals in mice [63].

Notably, reading speed and reading accuracy appear to be affected by PUFAs in different ways. EPA and DHA have an overall beneficial effect on reading speed, with the former acting as a predictor and the latter as a moderator. Therefore, it is plausible that the direct effect of EPA (which, in the presence of DHA and ALA, improves reading speed), not linked to auditory–phonological processes, reflects the use of anticipation strategies that preferentially rely on lexical decoding. In the presence of DHA (perhaps acting on visual efficiency and visual attention, as suggested by correlations and by the literature, see [19], or perhaps improving the efficiency of the processing of auditory stimuli, as shown by [64] for some fish species, or on a more general improvement in neuronal signaling), these strategies could be better integrated with information provided by the sublexical route. The indirect effects of EPA mediated by AP also contribute to enhancing speed, possibly through more efficient use of the sublexical route of reading. These hypotheses are supported by an additional analysis of the correlations between PUFA measures and reading errors and time subdivided into words and nonwords (reported in Table A4 in the Appendix A). Indeed, the favorable effect of ALA (in the AA/ALA ratio) on reading speed is significant for words (for which reading speed could take advantage of passing from the sublexical to the lexical route) but not for nonwords (which cannot be read through a completely lexical strategy), while the opposite is true for DHA. Finally, the contrasting effects found for EPA on reading speed and accuracy could also be explained by a form of trade-off phenomenon, with errors decreasing also as a result of slower reading. Unfortunately, little support can be obtained from the literature on molecular processes, which has focused very little on the cognitive effects of LCPUFAs in cognitive and learning processes and very rarely in humans. Among the rare studies showing the effects of LCPUFAs on auditory processing efficiency, most highlight the role of DHA but not of EPA (see Rahimi and coll.’s review [55], which, however, also addresses the potentially positive role of ALA). Among studies on reading and dyslexia, Cyhlarova and colleagues [52] highlight the role of EPA (and seem to disregard DHA, although it was listed among analyzed omega-3 LCPUFAS). However, Kairaluoma [65] did not find any effect of EPA supplementation in children with dyslexia.

The positive effect of both omega-3 LCPUFAs on writing accuracy confirms what has already been described regarding reading speed. Specifically, the effect of EPA proceeds essentially through AP and thus through more precise (de)coding, whereas the effect of DHA acts on EPA’s direct effect on the accuracy of the spelling process. In this case, the benefits of omega-3 LCPUFAs could be related to better integration of both routes for more efficient writing.

Among the limitations of this study, we can list the relatively small sample size, not allowing multiple interactions to be studied and disentangled, and the cross-sectional design, constraining the interpretation of underlying mechanisms to a rather speculative level. Longitudinal data offering the possibility to analyze the links between changes in omega-3 LCPUFA levels and related changes in specific neuropsychological tests as well as in learning abilities will allow stronger conclusions to be drawn from the data. Larger and more homogeneous samples will also allow for more powerful and more conservative analyses and criteria to be applied in order to avoid possible type I errors. Furthermore, the availability of more numerous samples could lower the probability of statistical model instability.

Further research into the biological mechanisms explaining the effects of omega-3 LCPUFAs on neurocognitive structures and functions is needed to open new pathways for intervention in learning disorders. Moreover, exploration of associations between LCPUFAs, reading/writing measures, and neuropsychological mechanisms should be conducted separately in the group of children with DD and in the group of TD children. Finally, the simultaneous analysis of dietary information will provide a more complete picture and help exclude the influence of potentially confounding factors.

## Figures and Tables

**Table 1:** Results of factor analysis emerged from “minimal residual” extraction with Oblimin rotation.

	Factors (n = 73)			
	1-AP	2-VP	3-VA	Unicity
Phonemic blending (errors)	0.644			0.539
Phonemic segmentation (errors)	0.812			0.356
Phonemic awareness—Nepsy (accuracy)	−0.532		0.274	0.555
RAN color speed (seconds)		0.749		0.430
RAN color accuracy (errors)				0.952
Visual search speed (seconds)		0.576		0.673
Visual search accuracy (errors)	0.419		0.421	0.600
Flanker effect (seconds)			−0.371	0.857
Flanker facilitation (seconds)				0.952
SAV congruency effect (seconds)			0.748	0.428
SAV rule complexity (seconds)			0.488	0.677
Pattern total (accuracy)	−0.284	−0.252		0.803
VSA left−right cue effect (seconds)			−0.390	0.800
Gratings accuracy		−0.335		0.747
Motion accuracy		−0.495		0.713

**Table 2:** Participants’ characteristics and performance profiles in reading/writing measures, neuropsychological tasks, and PUFA levels. Results expressed as raw scores.

		Mean (SD) N = 73	Range
Participants’ characteristics	Age (years)	10.87 (1.48)	8.17–14.17
	IQ	102.89 (12.04)	83–128
Reading/writing measures	General reading errors	8.49 (6.96)	0–38.5
	General reading time (seconds)	142.81 (74.35)	55–498.5
	General writing errors	4.66 (3.51)	0–13
Neuropsychological measures	Phonemic blending (errors)	3.32 (3.33)	0–12
	Phonemic segmentation (errors)	2.14 (2.34)	0–12
	Phonemic awareness—Nepsy (accuracy)	44.86 (3.76)	35–52
	RAN color speed (seconds)	89.04 (19.98)	57.63–178.01
	RAN color errors	0.73 (1.37)	0–6
	Visual search speed (seconds)	21.03 (8.23)	10.20–51.75
	Visual search errors	0.34 (0.67)	0–3
	Flanker effect (seconds)	93.10 (102.41)	−189–476
	Flanker facilitation (seconds)	−28.42 (110.05)	−329–336
	SAV congruency effect (seconds) ^1^	1424.88 (422.81)	429.71–2483.19
	SAV rule complexity (seconds) ^1^	352.34 (326.67)	−416–1014
	Pattern total (accuracy)	17.44 (4.86)	8–24
	VSA left−right cue effect (seconds)	6.49 (102.36)	−431–375.70
	Gratings accuracy (%)	80.59 (13.93)	33.33–100
	Motion accuracy (%)	66.97 (12.80)	25–88–64
Neuropsychological factors	AP	−0.168 (0.894)	−1.38–3.06
	VP	−0.102 (0.856)	−1.40–3.13
	VA	0.032 (0.855)	−1.59–2.59
Omega-3 LCPUFA levels	EPA	0.28 (0.20)	0.01–0.89
	DHA	1.55 (0.54)	0.45–2.97
Other PUFA levels	AA/ALA	70.64 (38.49)	12.81–244.50

^1^ N = 73.

**Table 3:** Mediation model for general reading errors, with AP as a mediator and age and omega-3 LCPUFA levels as covariates. Significant results in bold (*p* < 0.05).

Type of Effect	Predictor	Estimate	SE	β	Z ^1^	*p*
Indirect	**EPA**	−5.77	2.33	−0.17	−2.48	**0.013**
	DHA	0.58	0.74	0.04	0.78	0.437
	Age	0.27	0.27	−0.13	−2.26	**0.024**
Component	**EPA ⇒ AP**	−1.65	0.57	−0.37	−2.88	**0.004**
	DHA ⇒ AP	0.16	0.21	0.10	0.79	0.431
	Age ⇒ AP	−0.17	0.07	−0.28	−2.55	**0.011**
	AP ⇒ General reading errors	3.51	0.72	0.45	4.90	**<0.001**
Direct	EPA	−4.45	3.69	−0.13	−1.21	0.228
	DHA	−0.99	1.28	−0.08	−0.78	0.436
	Age	−1.84	0.43	−0.39	−4.30	**<0.001**
Total	**EPA**	−10.33	4.00	−0.30	−2.58	**0.010**
	DHA	−3.38	1.46	−0.03	−0.26	0.796
	Age	−2.44	0.47	−0.52	−5.16	**<0.001**

^1^ N = 73.

**Table 4:** Mediation model for general reading time, with AP as a mediator and age and omega-3 LCPUFA levels as covariates. Significant results in bold (*p* < 0.05).

Type of Effect	Predictor	Estimate	SE	Β	Z ^1^	*p*
Indirect	**EPA**	−54.23	23.39	−0.15	−2.32	**0.020**
	DHA	5.42	7.01	0.04	0.77	0.440
	Age	−5.64	2.64	−0.11	−2.14	**0.033**
Component	**EPA ⇒ AP**	−1.65	0.57	−0.37	−2.88	**0.004**
	DHA ⇒ AP	0.16	0.21	0.10	0.79	0.431
	Age ⇒ AP	−0.17	0.07	−0.28	−2.55	**0.011**
	AP ⇒ General reading time	32.96	8.43	0.40	3.31	**<0.001**
Direct	EPA	54.25	43.43	0.15	1.25	0.212
	DHA	−26.53	15.09	−0.19	−1.76	0.079
	Age	−17.48	5.03	−0.35	−3.47	**<0.001**
Total	EPA	−1.43	44.96	−0.004	−0.03	0.975
	DHA	−20.54	16.39	−0.15	−1.25	0.210
	Age	−23.14	5.31	−0.46	−4.36	**<0.001**

^1^ N = 73.

**Table 5:** Moderated mediation model for general reading time, with AP as a mediator, DHA as a moderator, and age and EPA as covariates. Components are only reported when significant indirect effects are present. The effects of age and its interactions are not reported. Significant results in bold (*p* < 0.05).

Moderator	Interaction		Estimate	SE	Β	Z ^1^	*p*
DHA	EPA:DHA ⇒ AP		1.49	0.82	0.21	1.83	0.067
	EPA:DHA ⇒ General reading time		−124.21	59.62	−0.21	−2.08	**0.037**
	DHA:AP ⇒ General reading time		−8.62	12.37	−0.06	−0.70	0.486
**Moderators level**	**Type**	**Effect**	**Estimate**	**SE**	**Β**	**Z** **^1^**	***p***
Mean − 1·SD	Indirect	EPA ⇒ AP ⇒ General reading time	−117.32	42.09	−0.31	−2.79	**0.005**
	Component	EPA ⇒ AP	−2.77	0.83	−0.62	−3.34	**<0.001**
		AP ⇒ General reading time	42.41	8.36	0.50	5.07	**<0.001**
	Direct	EPA ⇒ General reading time	157.21	64.51	0.41	2.44	**0.015**
	Total	EPA ⇒ General reading time	50.22	66.69	0.14	0.75	0.451
Mean	Indirect	EPA ⇒ AP ⇒ General reading time	−74.06	27.50	−0.20	−2.69	**0.007**
	Component	EPA ⇒ AP	−1.96	0.58	−0.44	−3.35	**<0.001**
		AP ⇒ General reading time	37.76	8.36	0.45	4.52	**<0.001**
	Direct	EPA ⇒ General reading time	90.25	44.96	0.24	2.01	**0.045**
	Total	EPA ⇒ General reading time	13.44	46.85	0.04	0.29	0.774
Mean + 1·SD	Indirect	EPA ⇒ AP ⇒ General reading time	−38.28	22.67	−0.11	−1.69	0.091
	Direct	EPA ⇒ General reading time	23.28	46.14	0.06	0.50	0.614
	Total	EPA ⇒ General reading time	−23.34	49.34	−0.06	−0.47	0.636

^1^ N = 73.

**Table 6:** Moderated mediation model for general reading time, with AP as a mediator, AA/ALA as a moderator, and age and EPA as covariates. Components are only reported when significant indirect effects are present. The effects of age and its interactions are not reported. Significant results in bold (*p* < 0.05).

Moderator	Interaction		Estimate	SE	β	Z ^1^	*p*
AA/ALA	EPA:AA/ALA ⇒ AP		0.00	0.01	−0.02	−0.15	0.885
	EPA:AA/ALA ⇒ General reading time		2.68	0.47	0.46	5.66	**<0.001**
	AA/ALA:PA ⇒ General reading time		0.21	0.19	0.08	1.08	0.280
**Moderators level**	**Type**	**Effect**	**Estimate**	**SE**	**β**	**Z** **^1^**	***p***
Mean − 1SD	Indirect	EPA ⇒ AP ⇒ General reading time	−32.85	17.67	−0.09	−1.86	0.063
	Direct	EPA ⇒ General reading time	−120.08	35.69	−0.33	−3.37	**<0.001**
	Total	EPA ⇒ General reading time	−162.74	39.99	−0.44	−4.07	**<0.001**
Mean	Indirect	EPA ⇒ AP ⇒ General reading time	−45.15	18.26	−0.12	−2.4	**0.013**
	Component	EPA ⇒ AP	−1.40	0.50	−0.32	−2.82	**0.005**
		AP ⇒ General reading time	32.16	6.25	0.39	5.14	**<0.001**
	Direct	EPA ⇒ General reading time	−17.39	28.04	−0.05	−0.62	0.535
	Total	EPA ⇒ General reading time	−63.83	31.14	−0.17	−2.05	**0.040**
Mean + 1SD	Indirect	EPA ⇒ AP ⇒ General reading time	−58.23	24.24	−0.15	−2.40	**0.016**
	Component	EPA ⇒ AP	−1.45	0.56	−0.33	−2.59	**0.010**
		AP ⇒ General reading time	40.08	6.25	0.46	6.41	**<0.001**
	Direct	EPA ⇒ General reading time	85.31	31.67	0.22	2.69	**0.007**
	Total	EPA ⇒ General reading time	35.08	35.11	0.10	0.99	0.318

^1^ N = 73.

**Table 7:** Mediation model for general writing errors, with AP as a mediator and age and omega-3 LCPUFA levels as covariates. Significant results in bold (*p* < 0.05).

Type of Effect	Predictor	Estimate	SE	Lower	Upper	β	Z ^1^	*p*
Indirect	**EPA**	**−3.65**	1.39	−6.38	−0.92	−0.21	−2.62	**0.009**
	DHA	0.36	0.47	−0.55	1.28	0.06	0.78	0.434
	Age	−0.38	0.16	−0.69	−0.07	−0.16	−2.37	**0.018**
Component	**EPA** **⇒ AP**	**−1.65**	0.57	−2.77	−0.53	−0.37	−2.88	**0.004**
	DHA ⇒ AP	0.16	0.21	−0.24	0.57	0.10	0.79	0.431
	Age ⇒ AP	−0.17	0.07	−0.30	−0.04	−0.28	−2.55	**0.011**
	AP ⇒ General writing errors	2.22	0.35	1.53	2.91	0.57	6.32	**<0.001**
Direct	EPA	−0.02	1.81	−3.56	3.53	−9.64 × 10^−4^	−0.01	0.993
	DHA	−1.20	0.63	−2.43	0.03	−0.19	−1.91	0.056
	Age	−0.64	0.21	−1.05	−0.23	−0.27	−3.03	**0.002**
Total	EPA	−3.99	2.14	−8.18	0.21	−0.23	−1.86	0.062
	DHA	−0.71	0.78	−2.24	0.82	−0.11	−0.91	0.364
	Age	−1.02	0.25	−1.51	−0.52	−0.43	−4.04	**<0.001**

^1^ N = 73.

## Data Availability

Data have been deposited at https://doi.org/10.5281/zenodo.15554494, but due to the ongoing main project (clinical trial n. 677) and to restrictions set by the Ethical Committee, it can be shared only upon written request to the corresponding author and under appropriate agreements.

## Abbreviations

The following abbreviations are used in this manuscript:

DD	developmental dyslexia
ADHD	attention-deficit hyperactivity disorder
AA/ALA	arachidonic acid/alpha linolenic acid
DHA	docosahexaenoic acid
EPA	eicosapentaenoic acid
LCPUFAs	long-chain polyunsaturated fatty acids
LTP	long-term potentiation
TNF	tumor necrosis factor
IL	interleukin
HPA	hypothalamic–pituitary–adrenal
AP	auditory processing
VP	visual–perceptual
VA	visual–attentional

## Appendix A

**Table A1:** Spearman partial correlations (N = 73) between reading/writing measures and the three neuropsychological factors (* *p* < 0.05, ** *p* < 0.01, *** *p* < 0.001).

	1	2	3	4	5	6
1. General reading errors	-					
2. General reading time (seconds)	0.538 ***	-				
3. General writing errors	−0.652 ***	0.542 ***	-			
4. Factor 1-AP	0.641 ***	0.534 ***	0.67 5***	-		
5. Factor 2-VP	0.239 *	0.440 ***	0.303 **	0.417 ***	-	
6. Factor 3-VA	−0.298 *	−0.257 *	−0.242 *	−2.69*	0.030	-

**Table A2:** Spearman partial correlations (N = 73) between PUFAs and reading/writing measures (* *p* < 0.05, ** *p* < 0.01, *** *p* < 0.001).

	1	2	3	4	5	6
1. EPA	-					
2. DHA	0.542 ***	-				
3. AA/ALA	−0.038	0.060	-			
4. General reading errors	−0.337 **	−0.266 *	0.115	-		
5. General reading time (seconds)	−0.235 *	−0.237 *	0.283 *	0.538 ***	-	
6. General writing errors	−0.357 **	−0.276 *	0.147	0.652 ***	0.542 ***	-

**Table A3:** Spearman partial correlations (N = 73) between PUFAs and the three factors (* *p* < 0.05, ** *p* < 0.01, *** *p* < 0.001).

	1	2	3	4	5	6
1. EPA	-					
2. DHA	0.542 ***	-				
3. AA/ALA	−0.038	0.060	-			
4. Factor 1-AP	−0.348 **	−0.163	−0.018	-		
5. Factor 2-VP	−0.238 *	−0.105	−0.011	0.417 ***	-	
6. Factor 3-VA	0.203	0.280 *	−0.123	−0.269 *	0.030	-

**Table A4:** Spearman partial correlations (N = 73) (partialling out the effect of age) between PUFAs and reading/writing abilities subdivided into the various subtests.

	DHA	EPA	AA	ALA	AA/ALA
Spearman rho, p					
Word reading errors	−0.244, **0.039**	−0.337, **0.004**	0.001, 0.995	−0.016, 0.896	0.037, 0.755
Nonword reading errors	−0.229, 0.053	−0.290, **0.014**	−0.000, 0.999	−0.174, 0.145	0.154, 0.196
Word reading time	−0.193, 0.105	−0.223, 0.060	0.054, 0.641	−0.289, **0.014**	0.302, **0.010**
Nonword reading time	−0.261, **0.027**	−0.207, 0.081	−0.007, 0.955	−0.210, 0.077	0.217, 0.067
